# Removal of benzene, toluene, xylene and styrene by biotrickling filters and identification of their interactions

**DOI:** 10.1371/journal.pone.0189927

**Published:** 2018-01-02

**Authors:** Dongqi Liao, Enze Li, Jianjun Li, Peiyuan Zeng, Rongfang Feng, Meiying Xu, Guoping Sun

**Affiliations:** 1 South China University of Technology, Guangzhou, China; 2 Guangdong Provincial Key Laboratory of Microbial Culture Collection and Application, Guangdong Institute of Microbiology, Guangzhou, China; 3 State Key Laboratory of Applied Microbiology Southern, Guangzhou, China; 4 Guangdong Open Laboratory of Applied Microbiology, Guangzhou, China; CAS, CHINA

## Abstract

Biotrickling filters (BTFs) are becoming very potential means to purify waste gases containing multiple VOC components, but the removal of the waste gases by BTF has been a major challenge due to the extremely complicated interactions among the components. Four biotrickling filters packed with polyurethane foam were employed to identify the interactions among four aromatic compounds (benzene, toluene, xylene and styrene). The elimination capacities obtained at 90% of removal efficiency for individual toluene, styrene and xylene were 297.02, 225.27 and 180.75 g/m^3^h, respectively. No obvious removal for benzene was observed at the inlet loading rates ranging from 20 to 450 g/m^3^h. The total elimination capacities for binary gases significantly decreased in all biotrickling filters. However, the removal of benzene was enhanced in the presence of other gases. The removal capacities of ternary and quaternary gases were further largely lowered. High-throughput sequencing results revealed that microbial communities changed greatly with the composition of gases, from which we found that: all samples were dominated either by the genus *Achromobacter* or the *Burkholderia*. Different gaseous combination enriched or inhibited some microbial species. Group I includes samples of BTFs treating single and binary gases and was dominated by the genus *Achromobacter*, with little *Burkholderia* inside. Group II includes the rest of the samples taken from BTFs domesticated with ternary and quaternary gases, and was dominated by the genus *Burkholderia*, with little *Achromobacter* detected. These genera were highly associated with the biodegradation of benzene series in BTFs.

## Introduction

Benzene, toluene, xylene and styrene (collectively referred to as BTXS) are usually generated during dismantling and incinerating process of electronic wastes [1]. The release of these compounds into ambient air can cause harmful effects on health and environment. According to the Clean Air Act Amendments (CAAA) amended by Environmental Protection Agency (EPA) of United States, these compounds are toxic to human beings [2]. For example, benzene is considered to be teratogenic, carcinogenic and mutagenic [3]. Long-term exposure of styrene can cause a variety of health problems such as headache, fatigue, weakness, depression, and peripheral neuropathy [4]. Although toluene and xylene have been not yet classified as carcinogens, the increase in rectal and colon cancer incidences has been reported among the exposed population [5]. Besides, these volatile organic compounds (VOCs) can react with nitrogen oxides in the presence of sunlight to produce ozone and peroxyacetyl nitrate, thus lower the air quality [6].

Several methods have been developed to treat VOCs-containing waste gases in recent decades, including adsorption, thermal incineration, wet scrubbing, catalytic oxidation and biofiltration, etc [7–11]. Among them, biofiltration has been successfully applied to remove single or mixed VOCs from waste gases due to its cost-effective and environmental-friendly advantages [12–14]. Since many industrial waste gases are emitted with multi-components, the assessment of comprehensive removal is crucial for the optimization of biofiltration process. Literatures revealed that when two or more VOCs were present, the degradation of one component could be either enhanced through co-metabolism, or inhibited though catabolic repression including competition of enzymes and depletion of electron acceptors [15, 16]. Specifically, some studies detected synergistic and inhibitory interactions among benzene, toluene, ethyl-benzene and xylene in biodegradation [17, 18]. However, the interactions among BTXS generated during pyrolysis of electronic wastes were rarely reported, especially those in biotrickling filters (BTFs).

Undoubtedly the microbial consortium in BTFs takes major responsibility for the removal of waste gases, and to optimize the performance, microbial communities of high diversity or those being pretreated (e.g. long-term domestication) are potential inoculums. In the present study, we employed four BTFs that were packed with polyurethane foam and were inoculated with pre-domesticated microbial communities to examine the elimination capacities (*EC*s) of BTXS in single or mixed forms. We also investigated the structures of microbial communities through High-throughput sequencing to correlate the removal efficiencies of different gas composition(s) to the corresponding microbial communities.

## Materials and methods

### Experimental set-up and inoculums

**Fig 1:**
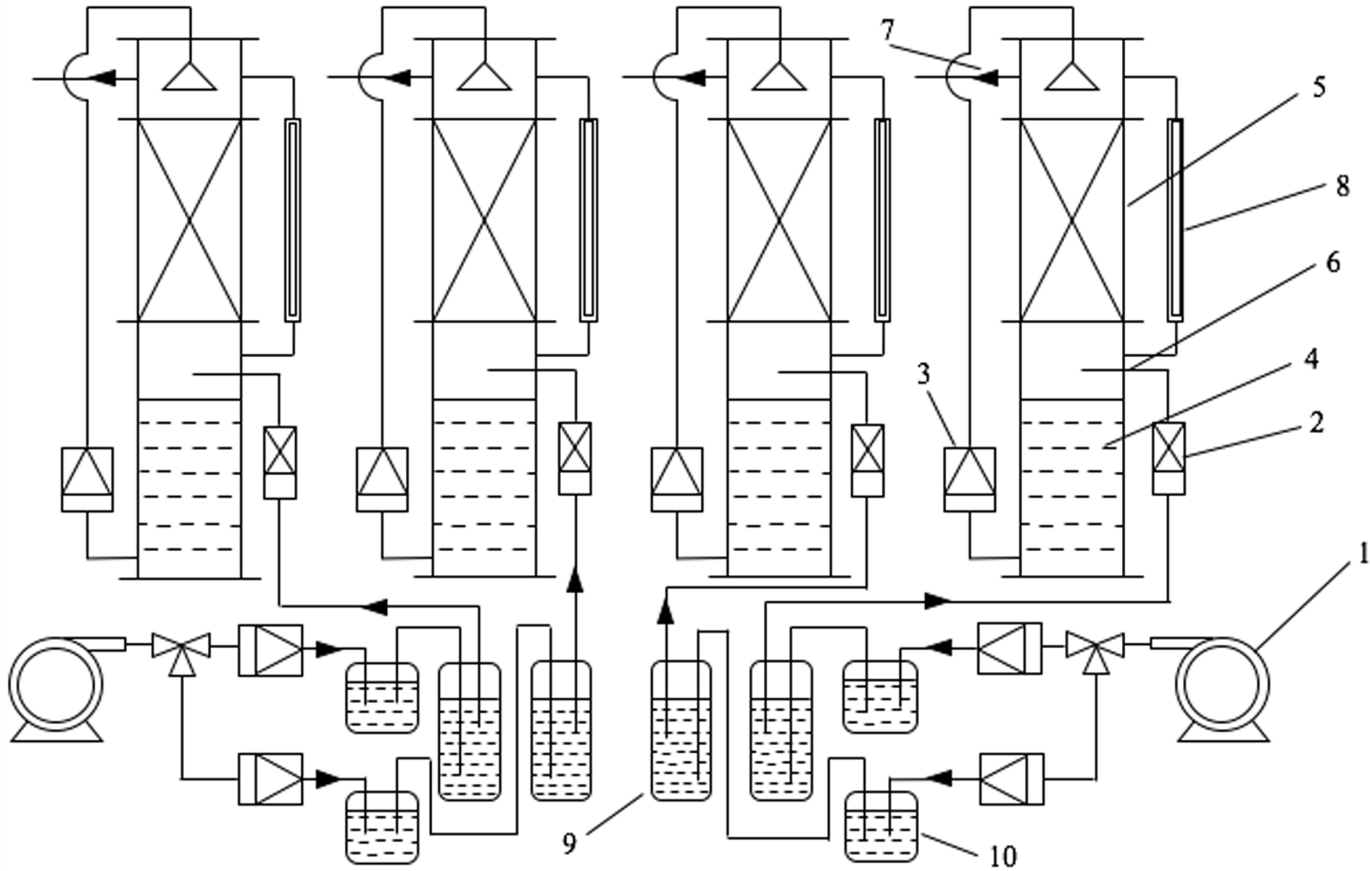
The laboratory-scale biofiltration system. 1. air compressor, 2. gas flow meter, 3. micro-syringe pump, 4. nutrient-holding tank, 5. packing material bed, 6. inlet gas sampling port, 7. outlet gas sampling port, 8. U-type manometer, 9. waste gas column, 10. humidification column.

Four laboratory-scale BTFs used in the present study were made of plexiglass, and operated in parallel (Fig 1). Each BTF comprised by one cylindrical packing column (inner diameter: 8 cm, height: 20 cm and effective packing volume: 1.0 L) and one nutrient holding column (4×4×20×3.14 cm) in a stacked configuration. The schematic diagram of four BTFs is showed in Fig 1. The packing column was filled with cubic polyurethane (PU) foam cubes (Dong Guan Material Factory, Guangdong, China) with a dimension of 1×1×1 cm. This packing material featured the porosity of 98%, particle size of 0.8±0.05 mm, bulk density of 0.015±0.03 g/cm^3^ and water holding capacity of 57.00±0.05 g/g, respectively.

Three microbial consortiums enriched from petroleum polluted soil were proved with the capability of degrading toluene, xylene and styrene, respectively. They were acclimated with toluene, xylene and styrene as carbon source, respectively over two years in our laboratory. In this study, three microbial consortiums with same OD600 (OD600 = 0.5) and volume (total 4 L) merged into one in order to keep the same biomass and microbial species in four biotrickling filters on the startup stage. They were firstly incubated in modified M9 medium at 30°C and 180 rpm for about 24 h, and then used as inoculums of the four BTFs. To keep the initial states of the four BTFs at close levels, all BTFs were inoculated with three microbial communities at equal amount (according to OD_600nm_). The nutrient solution was continuously sprayed on the packing materials using a peristaltic pump (Longer, Hebei, China) at a flow velocity of 0.5 m/min, and was renewed periodically. The modified M9 medium contained (per liter) 0.005 g/L NaH_2_PO_4_, 0.600 g/L Na_2_HPO_4_·12H_2_O, 0.020 g/L CaCl_2_, 2.000 g/L KNO_3_, 0.250 g/L MgSO4·7H_2_O, 0.005 g/L FeSO_4_·7H_2_O, 1 mL Vitamins and 1 mL trace elements. The pH of the nutrient solution was adjusted to about 7.2 by addition of 0.1 M NaOH.

For generating the waste gases containing BTXS, compressed air produced by an air compressor was split into a major and minor air streams. Liquid BTXS mixture was injected into the minor air stream via a syringe pump (model number: FP-SYP-01, Shanghai, China), and finally converged to the major airstream in a chamber. Synthetic polluted gases were introduced into BTFs from the bottom of packed column. The required concentration of gases was obtained by adjusting the injection rate of pure BTXS solution and the gas flow rate. Four BTFs were firstly supplied with 40 g/m^3^h of inlet loading of TXS mixture in equimolar amount. The flow rate and empty bed residence time (*EBRT*) was set at 0.12 m^3^/h and 30 s throughout the experiment. The experimental procedure was listed in Table 1. For testing the elimination capacities of BTFs for gases containing single or multiple components, inlet concentrations were progressively increased according to experimental procedure.

**Table 1:** Experimental procedures and conditions of biofiltration.

Stage No.Ⅰ: acclimatization			
components	Inlet concentration (g/m^3^)	Inlet Loading (g/m^3^h)	Time (day)
T+X+S	0.33	40	15
**Stage No.Ⅱ: treatment of single gas**			
toluene	0.33–6.67	40–800	10
styrene	0.33–5.00	40–600	
xylene	0.33–4.17	40–500	
benzene	0.17–3.75	20–450	
**Stage No. Ⅲ: treatment of binary gas**			
T+B	0.33–6.25	40–750 (20+20)-(375+375)	20
T+S	0.33–4.17	40–500 (20+20)-(250+250)	
T+X	0.33–5.00	40–600 (20+20)-(300+300)	
S+B	0.33–3.33	40–400 (20+20)-(200+200)	
S+T	0.33–2.08	40–250 (20+20)-(125+125)	
S+X	0.33–2.5	40–300 (20+20)-(150+150)	
X+B	0.33–2.08	40–250 (20+20)-(125+125)	
X+T	0.33–1.67	40–200 (20+20)-(100+100)	
X+S	0.33–2.08	40–250 (20+20)-(125+125)	
**Stage No. Ⅳ: treatment of tertiary gas**			
T+S+X	0.33–1.5	40–180	7
**Stage No. Ⅴ: treatment of quaternary gas**			
T+S+X+B	0.33–1.17	40–140	7

B: benzene; T: toluene; X: xylene; S: styrene.

### Analytical method

Gas samples were collected from an inlet gas port and an outlet gas port using 1 L sample bags (Tedlar, USA). Gas concentrations were analyzed gas chromatography (GC 5890, HP) equipped with a flame ionization detector, and an HP-5 chromatographic column (25 mm, 0.32 mm and 0.52 μm).The oven temperature, column temperature and the detector were set at 60°C, 180°C and 250°C, respectively. Nitrogen was used as the carrier gas at a flow rate of 1.2 mL/min. Injection volume was 50 μL. The linear range of the method was 1–1000 ppm (r^2^ = 0.9995). The pH of the nutrient solution was measured using an S40 SevenMulti^TM^ (Beckman, USA). The ambient temperature was measured by a thermometer (0–100°C).

### Microbial community structure analysis

High-throughput sequencing of 16S rRNA amplicon was used to investigate the microbial communities in the four BTFs. After characterizing the removal efficiency of each gas combination, triplicate packing materials (10 g, wet weight) from the top, middle and bottom levels of each BTF were collected sterilely, mixed with 10 mL phosphate buffer (2.70 mM KCl, 137 mM NaCl, 1.40 mM KH2PO4, 4.30 mM Na2HPO4; pH 7.3), and vortexed for 30 min. This was for the detachment of biofilm in order to release the microbial communities trapped inside. The cell-free packing materials were then discarded and the liquid phase containing biofilm and planktonic cells was centrifugated (8,000×g, 10 min) [19].

The genomic DNA (gDNA) was extracted from the pellets using a DNA isolation kit (PowerSoil®, MO BIO Laboratories, Inc) according to the manufacturer’s instruction. The gDNA quality and density were examined using a NanoDrop spectrometer. The double-barcoded primers 515F (5′-GTG CCA GCM GCCGCG GTA A-3′) and 806R (5′-GGACTACHVGGGTWTCTAAT-3′) were used to amplify the V4 hypervariable region of the 16S rDNA [20, 21]. The PCR mixture (50 μl) contained 20 ng of soil gDNA, 5 μl 10×EasyPCR buffer, each primer at 0.5 μM, each dNTP at 20 μM and 1 U of TransStart Fast Pfu DNA Polymerase (TransGen, China). The PCR program included an initial denaturation at 94°C for 1 min, followed by 32 cycles of 94°C for 20 s, 57°C for 25 s and 68°C for 45 s, and a final extension at 68°C for 10 min. PCR products were examined on a 1.5% w/v TAE-agarose gel, from where the bands with expected size were excised and recovered. The final quality control before library construction was guaranteed by Qubit 3.0 (to specifically determine the concentration of double stranded DNA) and Nanodrop (to exclude protein or RNA contamination). The fragment library was constructed with VAHTS^TM^ Nano DNA Library Prep Kit for Illumina (Vazyme Biotech Co.,Ltd).

The sequencing platform was Illumina^®^ MiSeq PE300. Paired-end reads were joined by FLASH (v2.2.00) and were processed using *Usearch* pipeline (v10.0.240) [22, 23]. Sequences having any one of the below conditions were filtered: (a) unidentifiable barcode (>1 base error) or primer (>3 base errors); (b) one or more “N” bases; (c) out of the range between 200~350 bp; (d) five or more consecutive bases with average Q-scores below 25; (e) singletons. Chimeric sequences were also filtered against the SILVA database (release 123). After quality control, the clean reads were used to construct the OTU table at 97% cutoff. The taxonomic assignment was performed with the RDP classifier (v2.12) at a 50% confidence level. Each sample was rarefied to the lowest read (10561) for statistical analysis, and beta-diversity analyses (PCA, PCoA). OTUs (≥ 0.1% relative abundance) were selected to compare the microbial communities of the four BTFs (heatmap). All statistical analyses were made with the R package.

### Evaluation of performance of BTFs and gas interaction indices

The removal performances of BTFs were evaluated by the following equations.

$$IL=\frac{C_{in}\;Q}{V}$$ (Eq 1)

$$EC=\frac{Q(C_{in}-C_{out})}{V}$$ (Eq 2)

$$RE=\frac{\left(C_{in}-C_{out}\right)}{C_{in}}\times 100$$ (Eq 3)

Where *IL* is the inlet loading rates, *RE* is the removal efficiency, *C* is the concentration, *C*_in_ is the inlet concentration, *C*_out_ is the outlet concentration, *Q* is the gas flow rate, and *V* is the packed volume.

The gas interaction indexes were used to assess the interaction, and were calculated according to the following equation, which is defined as [24]

$$Gas\;interaction\;index=\frac{A_{B}-A}{A}$$ (Eq 4)

Where A is the *EC* of compound A at 90% *RE*, AB is the *EC* of compound A in the presence of compound B at 90% *RE* of A. A negative index value indicates an inhibitory effect, while a positive index value shows a synergistic interaction effect. Specific value reflects the degree of the negative or positive interaction.

## Results and discussion

### Startup of four biotrickling filters

Four biotrickling filters (BTFs) for the experiments were inoculated with three microbial consortiums mixed in equal ratio, which were pre-acclimated to toluene, xylene or styrene as sole carbon source, respectively. All BTFs were operated under identical conditions. The gas flow rate was set at 0.12m^3^/h, resulting a 30 s of empty bed residence time. 40 g/m^3^h of inlet loading of mixed gases containing equal amounts of toluene, xylene and styrene were supplied to each BTF. After the startup, removal efficiencies (*RE*s) of pollutants gradually increased in all BTFs. Total *RE*s at 90% of removal efficiency were recorded, and used to evaluate the removal performances.

### Elimination capacities of single pollutant

**Fig 2:**
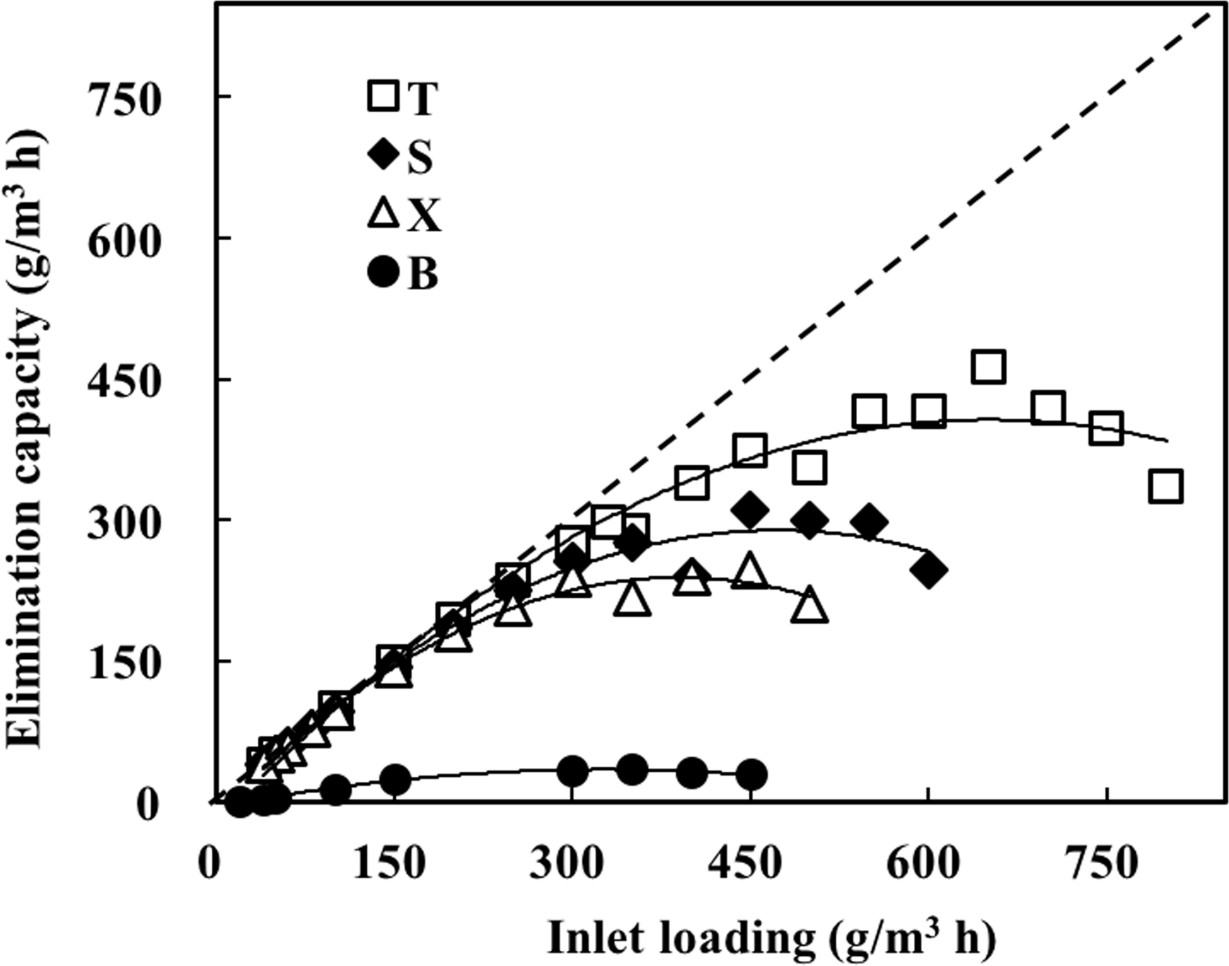
Elimination capacities of single toluene, styrene, xylene and benzene with inlet loadings. B: benzene; T: toluene; X: xylene; S: styrene.

For determining the elimination capacity (*EC*) of BTF for each pollutant, four BTFs (BTF1-BTF4) were operated in parallel, and were supplied with gases containing toluene, styrene, xylene or benzene, respectively. Elimination capacity of each BTF increased for all pollutants with increased inlet loading. Fig 2 showed that removal efficiencies for all BTFs begun to decrease when inlet loading reached a certain value. The *EC* obtained at 90% of RE was recorded to assess the removal performance of BTF on each pollutant. Results showed that *EC*s observed at 90% of *RE* for toluene, styrene and xylene were 297.02, 225.27 and 180.75 g/m^3^h, respectively. Corresponding inlet loadings were 330, 250 and 200 g/m^3^h, respectively. Toluene is among the most easily degradable pollutant, followed by styrene and xylene. Several other studies on microbial degradation of TXS compounds concluded the similar results [25,26]. No obvious removal was observed for benzene with inlet loadings from 20 to 450 g/m^3^h during this experimental phase. Possible reason for this can be attributed to the microbial consortium that formed within the BTF4. As mentioned above, the three consortiums had been kept for a long time in three desiccators those filled with gases containing toluene, xylene or styrene, respectively. These consortiums needed times to adapt the benzene. Furthermore, benzene has relatively more stable chemical structure than others despite the fact that these aromatic compounds are similar in chemical structure [27].

### Removal performances of BTFs on various binary gases

**Fig 3:**
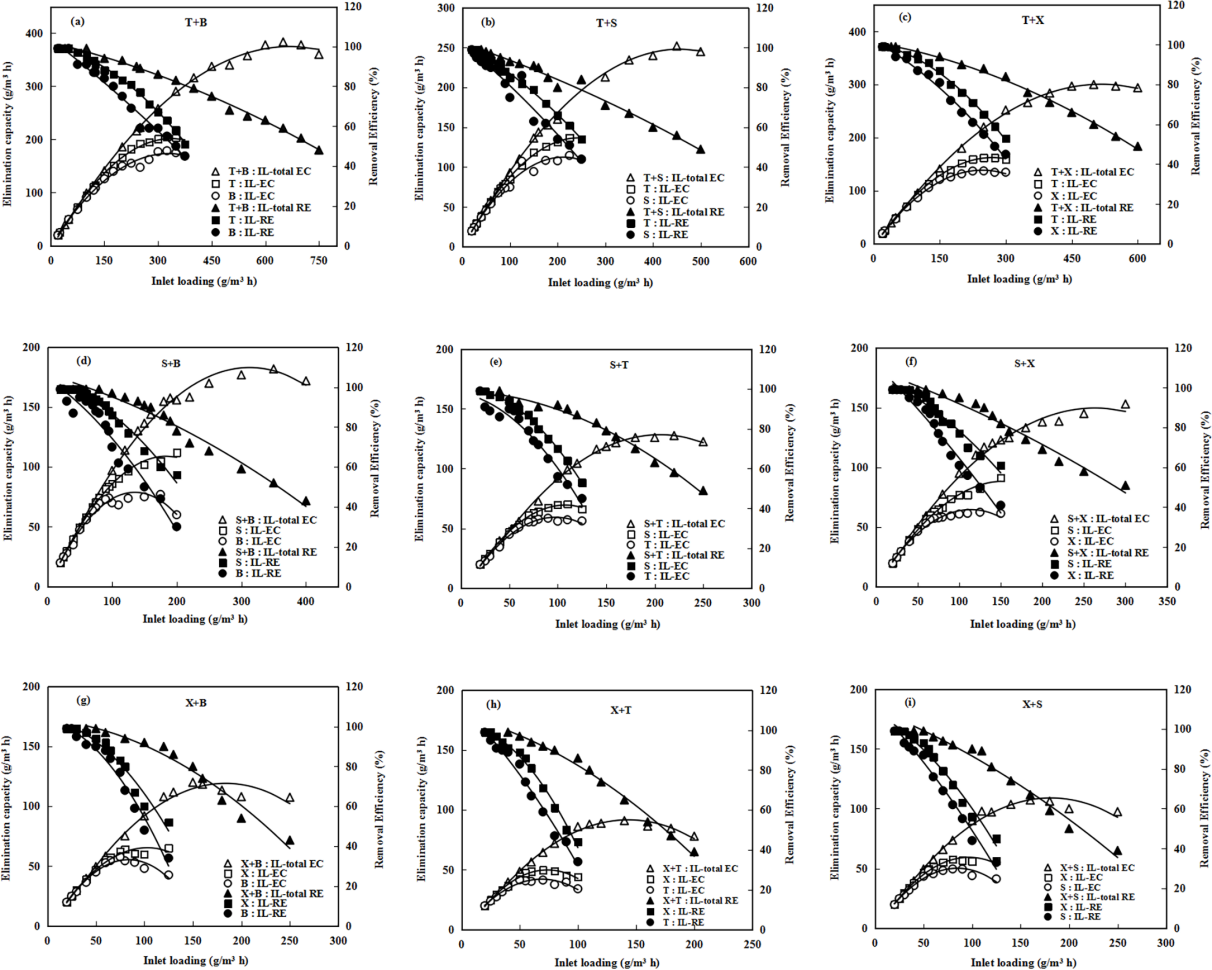
Comparison of elimination capacities and removal efficiencies of binary gases with inlet loadings. B: benzene; T: toluene; X: xylene; S: styrene.

Since most of industrial waste gases contain multiple components, the identification of interaction thus is important for improving the removal performance of BTF treating complex waste gases. During the experimental stage Ⅲ in Table 1, different binary pollutants taken turns to be supplied into three BTFs (BTF1, BTF2 and BTF3) according to the experimental procedure. The fourth BTF (BTF4) was performed to test the interaction among tertiary and quaternary pollutants. To compare with single pollutant, total *EC* for binary pollutant at 90% of total *RE* was also recorded. The comparison of *EC*s was done to characterize the attributes of interaction, while the comparison of *RE*s between each component of the binary pollutants gases allowed showing that which component dominated the interaction. Data on *EC*s and *RE*s were plotted in Fig 3. As for the BTF1, when toluene and benzene binary gases were supplied, total *EC* observed at total RE of 90% was 216.58 g/m^3^h, which is far less than the *EC* value of single toluene(297.02 g/m^3^h), indicating that adding benzene largely lowered the overall removal performance of the BTF (Fig 3A). Garcia-Pena et al reported that the toluene degradation rate (0.27 mg/l^3^h) after adding benzene was lower than the rate obtained with single toluene (0.37 mg/l^3^ h) by the fungus *Paecilomyces variotii* [28]. However, *RE*s for benzene and toluene in this scenario were 87% and 93%. So it can be concluded that toluene greatly enhanced the removal of benzene since that benzene was hardly removed alone by the BTF1. Dou et al reported that the amendment of toluene could stimulate benzene degradation by enriched BTEX-degrading bacteria [17]. When toluene was treated along with xylene or styrene in the binary form by BTF1, total *EC* for the two binary gases at 90% of total *RE* was 180.33 and 144.68 g/m^3^h, respectively (Fig 3B and 3C). The reduction of *EC*s compared to single component gas means that inhibition effects also occurred in the two binary gases. Shim et al reported the similar results about removal of benzene, toluene, xylene and methyl tert-butyl ether by using a biotrickling filter [24]. The *RE*s of xylene and styrene dropped more rapidly with increased total inlet loading rates compared to toluene, indicating that xylene and styrene were inhibited more than toluene.

The BTF2 was employed to assess the effects of benzene, toluene or xylene on the styrene removal. The total *EC*s at total 90% of *RE* were also compared. It can be found from Fig 3D, 3E and 3F) that total *EC* values decreased for all binary gases. Among them, binary gas composed by styrene and toluene had poorest removal in the BTF2 (Fig 3E). Total *EC* for this binary gas at 90% of *RE* was only 99.32 g/m^3^h, which was lower than others (143.97 g/m^3^h for styrene and benzene, and 117.06 g/m^3^h for styrene and xylene). The binary gas of styrene and toluene was also treated in the BTF1. However, it is obvious that the BTF1 showed a superior removal for this binary gas compared to BTF2 (144.68 g/m^3^h versus 99.32 g/m^3^h). During the experimental stageⅡ, BTF1 and BTF2 were once supplied with toluene and styrene, respectively. So microbial community formed inside BTF1 may differ from that in BTF2. More diversely functional microbes may be developed within the BTF1, and therefore enhanced the removal performance of the BTF1 for complex gases. Best removable binary gas in the BTF2 was the combination of styrene and benzene (Fig 3D). The *EC* of BTF2 for benzene at 90% *RE* was 69.62 g/m^3^h, closed to the corresponding *EC* of styrene (74.41 g/m^3^h). It is evident that benzene removal was also enhanced in the present of styrene. This is consistent with the observation from the BTF1 treating toluene and benzene binary gases. It is most likely that microbes degraded the benzene through the co-metabolisms [17].

Xylene was co-treated along with benzene, toluene or styrene in the BTF3 (Fig 3G, 3H and 3I). The total *EC*s at total 90% of *RE* obtained by BTFs for each binary gas were lower than those observed in the BTF1 and the BTF2 (108.12 g/m^3^h for xylene and benzene, 72.43 g/m^3^h for xylene and toluene, and 90.07 g/m^3^h for xylene and styrene). However, the trends of drop in *RE*s of xylene in BTF3 were weaker than other component in all binary gases. Alvarez et al reported that enhanced degradation of xylene occurred in the presence of toluene and benzene by *pseudomonas* sp. strain CFS-215 incubations [29]. Microorganisms inside the BTF3 more likely belong to xylene-degrader since the BTF3 was supplied only with xylene before binary test. This also can explain similar phenomenon occurred in the BTF1 and the BTF2.

### Removal performance of the BTF4 for ternary and quaternary gases

**Fig 4:**
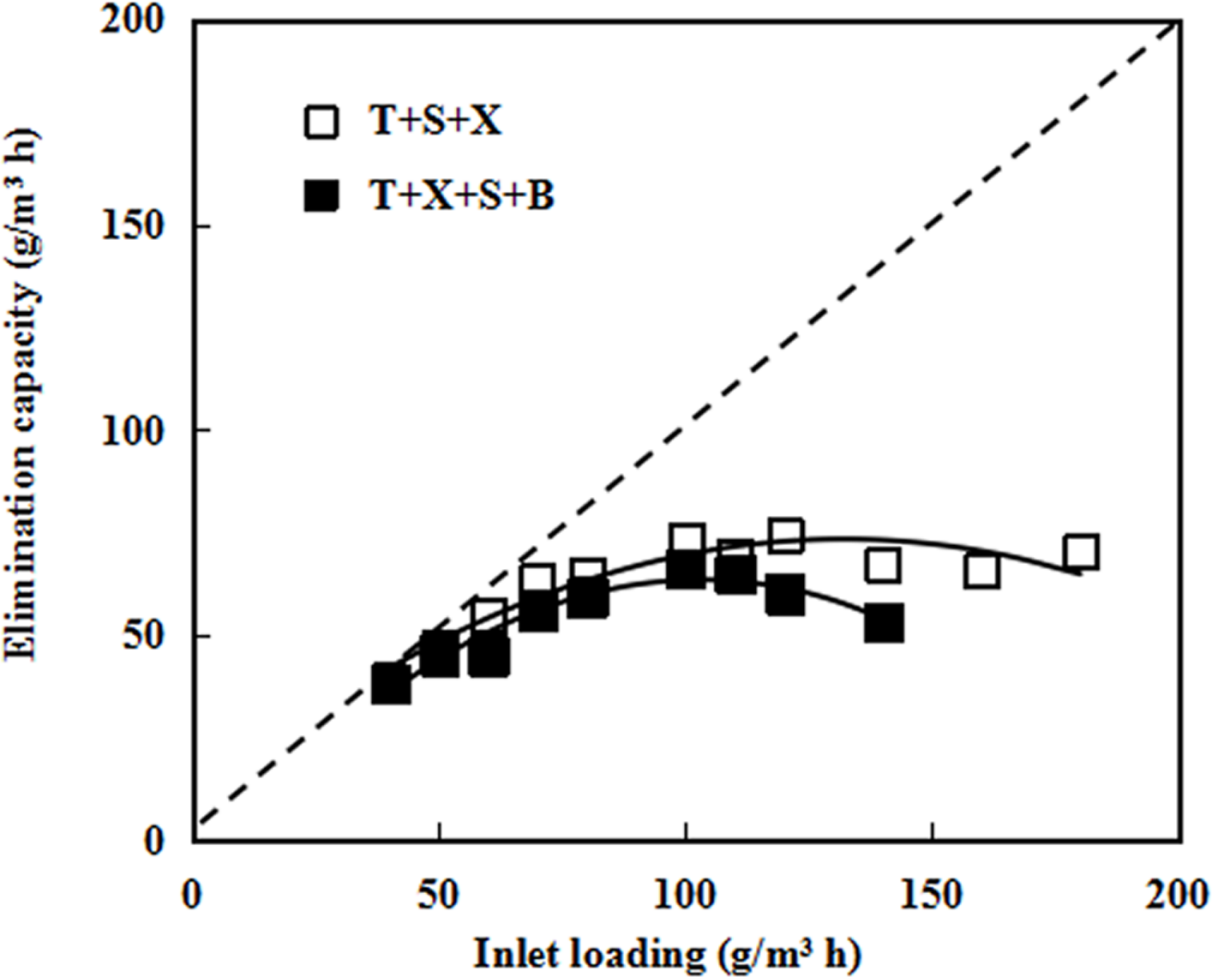
Elimination capacities of ternary (TXS) and quaternary (BTXS) mixture with inlet loadings. B: benzene; T: toluene; X: xylene; S: styrene.

Ternary (TXS) and quaternary (BTXS) gases were treated in the BTF4. Results showed that removal performance of the BTF4 seemed to decrease with the increase of gaseous complexity. As shown in Fig 4, total *EC* at 90% of total *RE* was 63 .25 g/m^3^h for ternary gases, and was only 45.18 g/m^3^h for quaternary gases. These ECs values only account for 20.83% to 62.50% of those observed with binary gases. In addition to the gaseous complexity, the difference between the compositions of microbial communities may be another crucial factor. Although the BTF4 was supplied with TXS mixed gases during startup stage as same as other reactors, only benzene was introduced into BTF4 during second stage. No obvious degradation of benzene was observed during the second stage, and that the co-metabolism of benzene and other pollutant in binary gases further showed the possible difference in microbial community among the four BTF [24]. Comparatively, benzene was among the most difficultly degraded pollutant followed by xylene, styrene and toluene. The similar results were concluded on microbial degradation of BTX compounds by Choi et al [30]. It is proved again that the degradation of benzene needed the presence of other pollutants. The interaction indexes between xylene and other pollutant were relatively high, showing that xylene more easily influenced the overall removal performance for mixed gases compared to other pollutant (Table 2).

**Table 2:** Gas interaction indexes between each two pollutants.

Gas interaction indices indicating the effects of BTXS on biodegradation of T, S or X in different mixture			
	BTF3	BTF2	BTF1
	X	S	T
**+B**	-0.69	-0.64	-0.58
**+T**	-0.78	-0.76	-
**+S**	-0.73	-	-0.71
**+X**	-	-0.72	-0.61

B: benzene; T: toluene; X: xylene; S: styrene; BTF: Biotrickling filter.

### Comparisons of gas interaction indices in various binary gases mixture

Elimination capacities of toluene, styrene and xylene at 90% removal efficiency were evaluated in binary mixture (S1 Table). The gas interaction indexes of various pollutant combinations were calculated and listed in Table 2. Results showed that all interaction indexes are negative, indicating inhibitory effect occurred in all cases regardless of bioreactor used. Interaction indexes observed in BTF1 ranged from 0.58 to 0.71, which were lower than those observed in BTF2 and BTF3. This means that inhibition effects occurred in BTF1 were not severe compared to BTF2 and BTF3 even for the same binary gases. For instance, the interaction index between toluene and styrene in BTF1 was 0.71, while increased to 0.76 in BTF2. Since operating conditions like gas flow rate, nutrient level, and inlet loadings were identical in both BTFs, so the difference in the microbial composition between the two BTFs may be the leading cause for their differential interaction effects [24]. Besides, different pollutants showed the different interaction effects. The interaction indexes between benzene and other pollutants were relatively lower than that of other binary gases. It is proved again that the degradation of benzene needed the presence of other pollutant components. The interaction indexes between xylene and other pollutant were relatively high, showing that xylene more easily influenced the overall removal performance for mixed gases compared to other pollutants.

### Microbial community structure analysis

**Fig 5:**
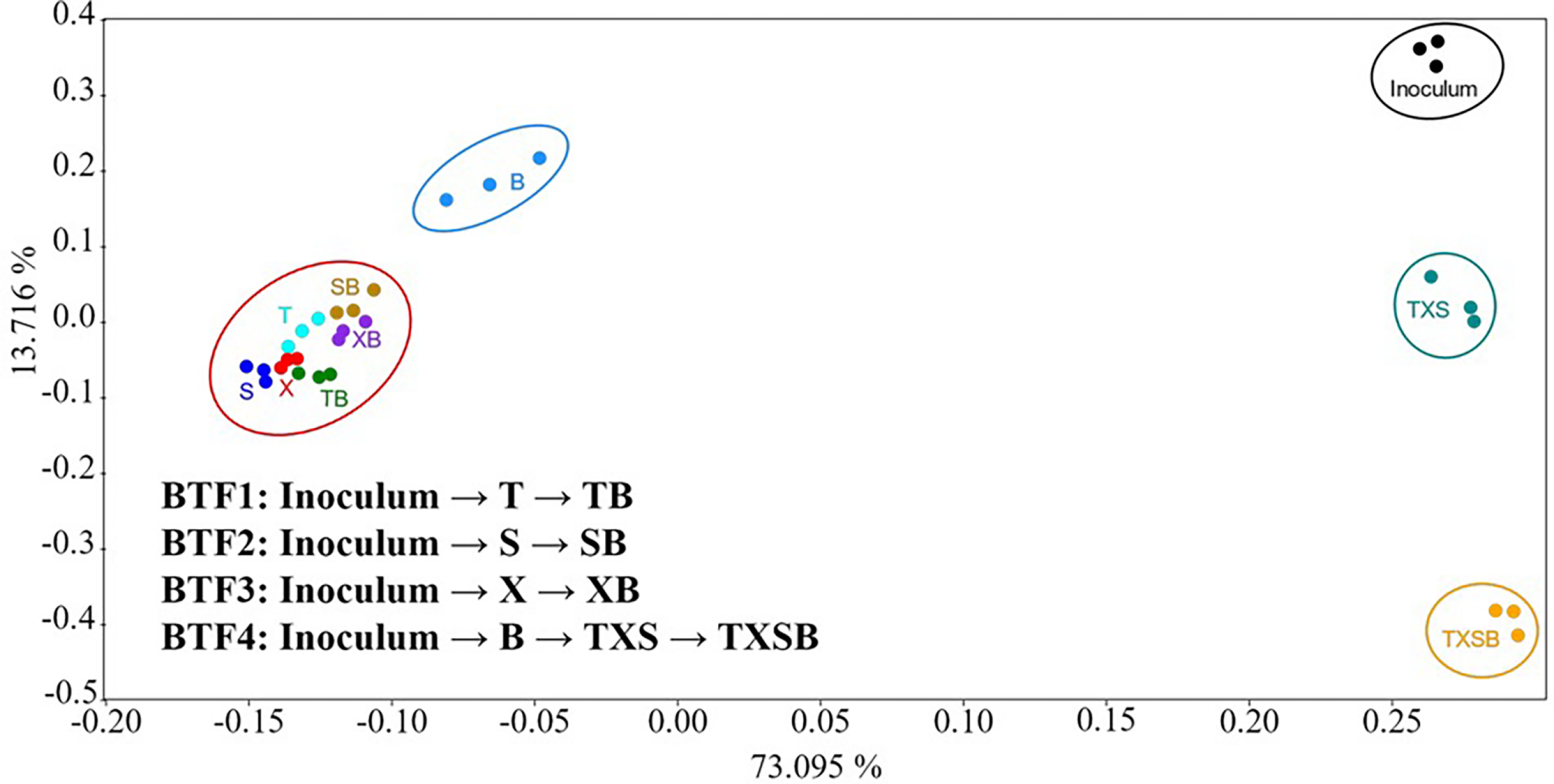
The plot of weighted principal coordinate analysis (PCoA) of the microbial communities based on the Bray-Curtis distance. B: benzene; T: toluene; X: xylene; S: styrene.

High-throughput sequencing of bacterial 16S rRNA gene generated 737k qualified reads across all samples with an average length of 265 bp. These reads were clustered into 1025 operational taxonomic units (OTUs) at a 97% cutoff. For statistical analyses, we rarefied the OTU table according to the lowest reads (10625 reads, sample TXS3). Triplicate samples from 9 treatments in the absence or present of benzene and the original inoculum (JZ1~3) were analyzed. Weighted principal coordinate analysis (PCoA) was performed based on the Bray-Curtis distance, and totally 86.81% variance of the OTU dataset was included in the first two principal coordinate axes (Fig 5). All samples fell into five groups. The inoculums, ternary (TXS) and quaternary gases (TXSB) were three distinct groups far separating from the other two. The single-component gases (T, S, X) and their benzene-containing binary gases (TB, SB, XB) were densely piled together. From the Fig 5, we summarized that (a) all treatments drifted away from the original communities (referring to the inoculums), especially the single and binary gases; (b) the benzene group was a distinct one from the rest single and binary treatments (T, S, X, TB, SB, XB), while the latter were closely piling together and formed a mixed group, circled in red; (c) the single gases (T, S, X) in the mixed groups were less affected when benzene was introduced. The reason why benzene group was separate from BTF1, 2 and 3 was because benzene was never introduced to the original communities in the pre-acclimation. That also explained the strong interference of benzene and a fast recovery we observed during the transition from Inoculum to benzene to TXS in BTF4.

**Fig 6:**
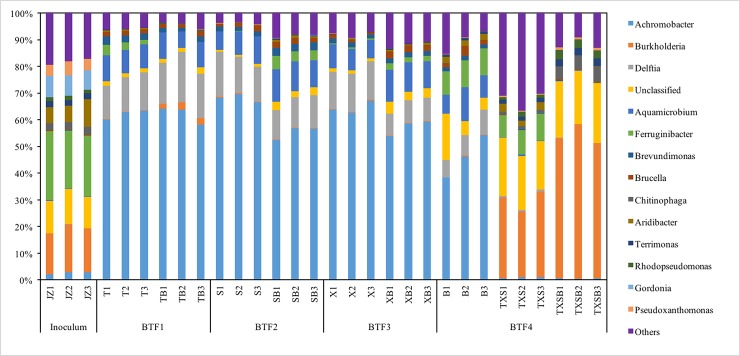
Microbial community structures of 30 samples from 9 treatments and the original inoculums. B: benzene; T: toluene; X: xylene; S: styrene; JZ: inoculum; BTF: Biotrickling filter.

The taxonomic assignment was performed against the RDP database at a 50% confidence, and we calculated the relative abundances at genus level to plot this histogram (Fig 6), and the corresponding heatmap was shown in S1 Fig. *Achromobacter* and *Burkholderia* were the two genera of most interest we located when looking into the detailed community structures at genus level. The result was significantly different from microbial community of degrading BTXE (E: Ethyl benzene) [31]. Dominant genera with relative abundance higher than 0.5% were plotted in Fig 6, and their changes were compared in an effort to better understand the removal behavior of all BTFs. *Achromobacter* was the most dominant genus in all single and binary gases regardless of individual treatment, ranging from 39.79% to 69.93%. In the rest samples including the Inoculum, ternary and quaternary gases however, it decimated to below 3% in relative abundance. The other genus of most interest, *Burkholderia*, had the opposite distribution pattern that it dominated the ternary and quaternary gases (from 27.75% to 58.32%) and occupied approximately one-sixth of the inoculums in relative abundance. In samples where *Achromobacter* dominated, *Burkholderia* was barely detectable (mostly less than 0.3%). These two genera seemingly had mutual antagonism or competed for some unique niches.

Several reports had demonstrated the aromatic degrading capacity and the genes involved in aromatic degrading enzymes in some species of the *Burkholderia* genus, such as the *tmoA* and *todC1* gene [32, 33]. Little was known about genus *Achromobacter* however, or at least very limited reports had associated *Achromobacter* with benzene and styrene degradation in biotrickling filters [34, 35]. When sole benzene was introduced to BTF4, it had a similar composition of dominant species compared to other BTFs. However, we noticed drastic shifts when BTF4 was further fed with ternary or quaternary VOCs, including the shrinking of *Achromobacter* and the enrichment of *Burkholderia*. We believe these shifts represented a complex network concerning microbial adaptive mechanisms to VOCs, but the exact associations correlating microorganisms to the specific biodegradation of benzene series were far from clear.

**Fig 7:**
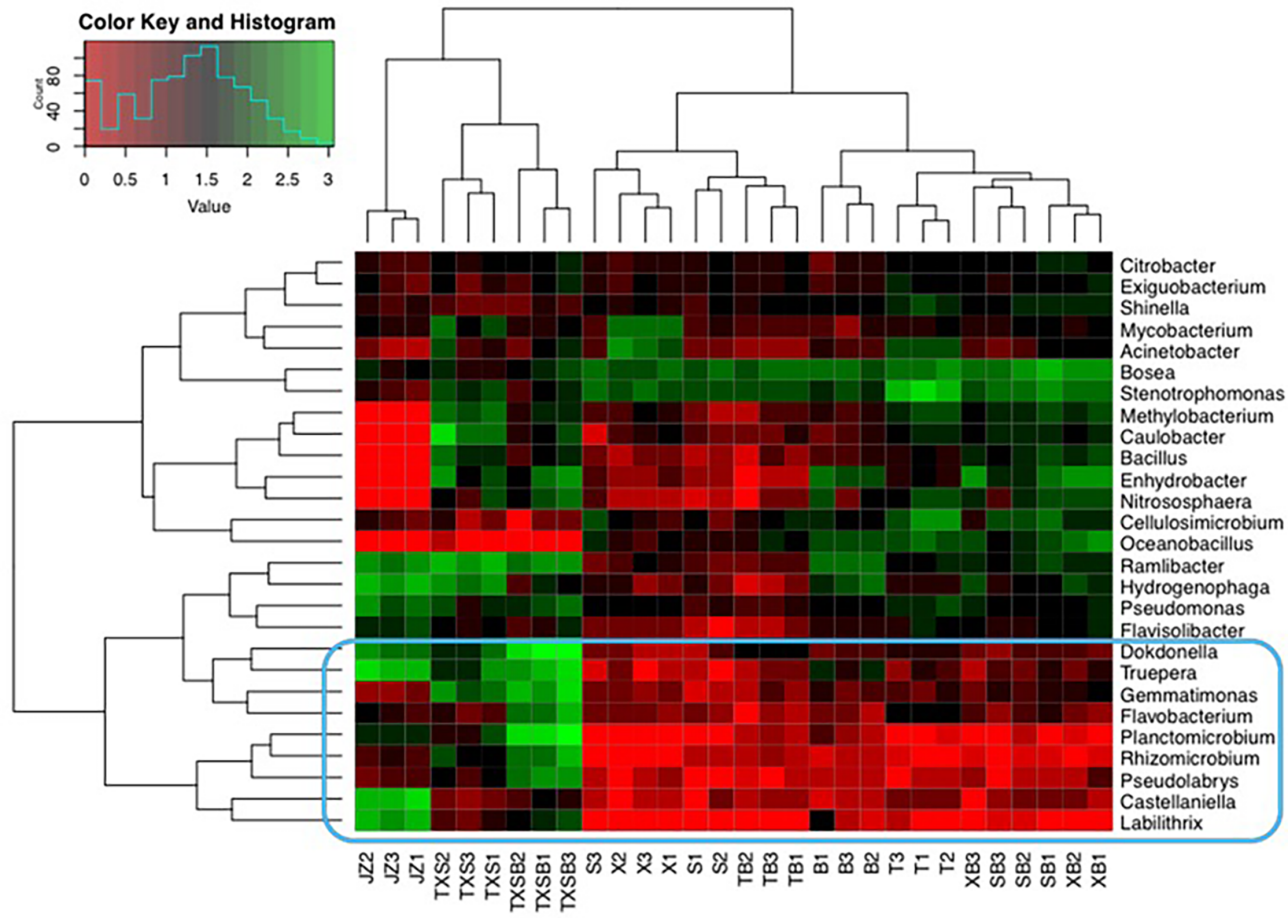
The heatmap of genera with relative abundances between 0.1% with 0.5%. B: benzene; T: toluene; X: xylene; S: styrene; JZ: inoculum; BTF: Biotrickling filter.

Species with low relative abundances (categorized in Others) also heavily impacted some of the samples like Inoculums (JZ), ternary (TXS) and quaternary VOCs (TXSB) groups. They were low in gross relative abundances but were considerably enriched in these three groups, especially in TXS (~ 45%). Therefore we attempted to locate a few candidates with significant differences between those three and the other seven treatments in the heatmap (Fig 7), which was plotted using rarefied OTU dataset (log10N). The genera in the blue square seemed to be the reason why “Others” took such large portions in JZ, TXS and TXSB.

## Conclusion

Industrial manufacturing is undoubtedly the top reason for VOC production, whose composition was highly complex. The biodegradation of one individual component may be promoted by microbial co-metabolism, or inhibited by enzymatic competition and catabolic regression when others are present [24]. Therefore, the interactions among various VOC components have to be taken into considerations because of their critical impacts on the performances of biotrickling filters. In this study we measured the elimination capacities and identified the interactions of BTXS by four BTFs, and found that the total removal decreased with increasing gas complexity. The elimination capacities at 90% removal efficiency of T, S and X appeared in single gases, but B was an exception that it was barely degradable unless other components were present, indicating its co-metabolic pattern in biodegradation. Comparison of microbial communities revealed that BTFs pre-adapted with target pollutants could develop the microbial communities with relatively high robustness and removal capacity. These finds will be contributed to provide new significant insights into biofiltration and improve the rational design of biotrickling filters for treatment of waste gases.

## Supporting information

S1 Fig The heatmap of genera with relative abundances beyond 0.5. B: benzene; T: toluene; X: xylene; S: styrene; JZ: inoculum; BTF: Biotrickling filter.
(TIF)

S1 Table Elimination capacities of toluene, styrene and xylene at 90% removal efficiencies in binary mixture. B: benzene; T: toluene; X: xylene; S: styrene; BTF: Biotrickling filter.
(DOCX)
