# Supplementary figures and images for: Removal of benzene, toluene, xylene and styrene by biotrickling filters and identification of their interactions
Source: PLoS One. 2018 Jan 2;13(1):e0189927. doi: 10.1371/journal.pone.0189927 (PMC5749713; doi:10.1371/journal.pone.0189927)

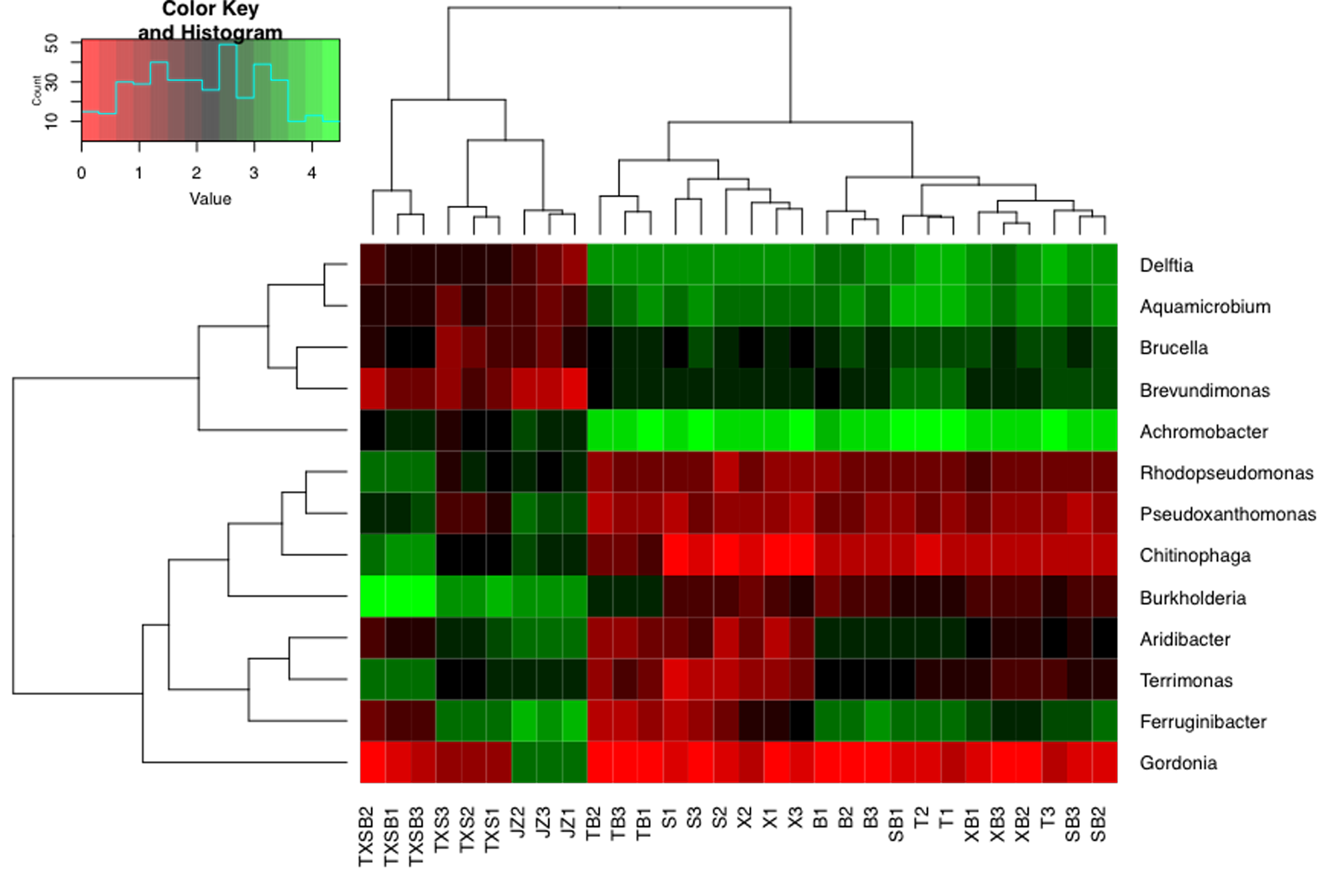

Supplement: S1 Fig — B: benzene; T: toluene; X: xylene; S: styrene; JZ: inoculum; BTF: Biotrickling filter. (TIF) [file pone.0189927.s001.tif]
