# Supplementary material for: Removal of benzene, toluene, xylene and styrene by biotrickling filters and identification of their interactions
Source: PLoS One. 2018 Jan 2;13(1):e0189927. doi: 10.1371/journal.pone.0189927 (PMC5749713; doi:10.1371/journal.pone.0189927)
Supplement: S1 Table — B: benzene; T: toluene; X: xylene; S: styrene; BTF: Biotrickling filter. (DOCX) [file pone.0189927.s002.docx]

**Supporting information**

**S1 Table.** **Elimination capacities of toluene, styrene and xylene at 90% removal efficiencies in binary mixture.**

|  | **BTF3** | | **BTF2** | | **BTF1** | |
| --- | --- | --- | --- | --- | --- | --- |
|  | **X** | | **S** | | **T** | |
|  | IL | EC | IL | EC | IL | EC |
| **B** | 62.50 | 56.25 | 90 | 81 | 140 | 126 |
| **T** | 45 | 40.50 | 60 | 54 | - | - |
| **S** | 55 | 49.50 | - | - | 95 | 85.50 |
| **X** | - | - | 70 | 70 | 130 | 117 |

B: benzene; T: toluene; X: xylene; S: styrene; BTF: Biotrickling filter.
